# Bilayered Retinoschisis during non-arteritic anterior ischemic optic neuropathy

**DOI:** 10.22336/rjo.2021.51

**Published:** 2021

**Authors:** Cagri Ilhan, Mehmet Citirik

**Affiliations:** * Hatay Education and Research Hospital, Hatay, Turkey; ** University of Health Sciences, Ankara Ulucanlar Eye Education and Research Hospital, Ankara, Turkey

**Keywords:** ischemic optic neuropathy, non-arteritic anterior ischemic optic neuropathy, optical coherence tomography, optic neuropathy, retinoschisis

## Abstract

**Objective:** To report an unusual optical coherence tomography (OCT) finding, bilayered retinoschisis formation in the macular region, during non-arteritic anterior ischemic optic neuropathy (NAION).

**Methods:** This is a case report of a patient with NAION and an unusual OCT finding, bilayered retinoschisis formation.

**Results:** A 62-year-old female presented with decreased visual acuity in her right eye for one year. The best-corrected visual acuity was determined as 20/ 160 and no abnormal anterior segment finding was found in the slit-lamb biomicroscopy. A pale optic disc was detected and a widespread peripapillary retinal nerve fiber layer (RNFL) thinning was observed in the fundus evaluation. In the macula OCT evaluation, retinoschisis and microcyst formations at the outer nuclear layer (ONL) in the central macula and additional retinoschisis formation at the inner nuclear layer (INL) in the temporal macula were observed.

**Conclusion:** As far as we know, this is the first report to demonstrate an unusual co-existence of NAION and retinoschisis that also has an unusual formation. The clinicians should be informed of the possible effects of NAION on the macula in the course of the disease.

## Introduction

Anterior ischemic optic neuropathy (AION) is a frequent reason for acute optic neuropathy in the 6th and 7th decades of life [**1**]. Different pathophysiological mechanisms are responsible for the arteritic (AAION) and non-arteritic AION (NAION) types of the disease. Short posterior ciliary artery occlusion plays an important role in the structural and functional changes in NAION [**2**]. The exact reason for this vascular insufficiency is not fully clarified, however, some systemic and local risk factors such as diabetes mellitus, systemic hypertension, hypercholesterolemia, atherosclerosis, smoking, stroke, and structurally crowding optic disc are known to be associated with the NAION [**3**,**4**]. Acute, painless, severe visual loss, visual field defects, relative afferent pupil defect, peripapillary hemorrhages, and optic disc swelling can be observed in the affected eye [**4**]. According to the outcomes of the Optic Nerve Decompression Treatment Trial, 1/ 3 of the subjects’ gain ≥ 3 lines after a 2-year follow-up, 30% of subjects lose ≥ 3 lines at the end of 2-year, and the rest of the subjects show no change in their visual acuity [**5**]. For the fellow eye, the risk of being affected is 10% within two years and 15-25% within five years [**6**].

Structural features of the optic disc and macula can be assessed by optical coherence tomography (OCT) through infrared light. The most important reason for OCT using in NAION is to quantitatively evaluate optic disc edema and peripapillary retinal nerve fiber layer (RNFL) loss [**7**,**8**]. This case report aimed to underline an unusual OCT finding, bilayered retinoschisis formation in the macula, during NAION. As far as we know, this case report demonstrates for the first time, an unusual co-existence of these two sight-threatening conditions. 

## Case report

A 62-year-old female complained of decreased visual acuity in her right eye. The visual loss persisted for the last 12 months. The patient had no history of ocular trauma, received ocular surgery, chronic ocular disease, or medication. Systemic hypertension was the only systemic disease and no history of smoking, alcohol, or other addiction. The best-corrected visual acuity was examined by a Snellen chart as 20/ 160 in her right eye and 20/ 20 in the left eye. Manifest refraction spherical equivalent was determined as < 2 D hyperopia in both eyes. The intraocular pressure values with the Goldmann applanation tonometer were 12 and 15 mmHg. No abnormal anterior segment finding was detected in the slit-lamb biomicroscopy. In the fundus evaluations, a pale optic disc was observed in the right eye while the left eye was completely normal. 

**Fig. 1:**
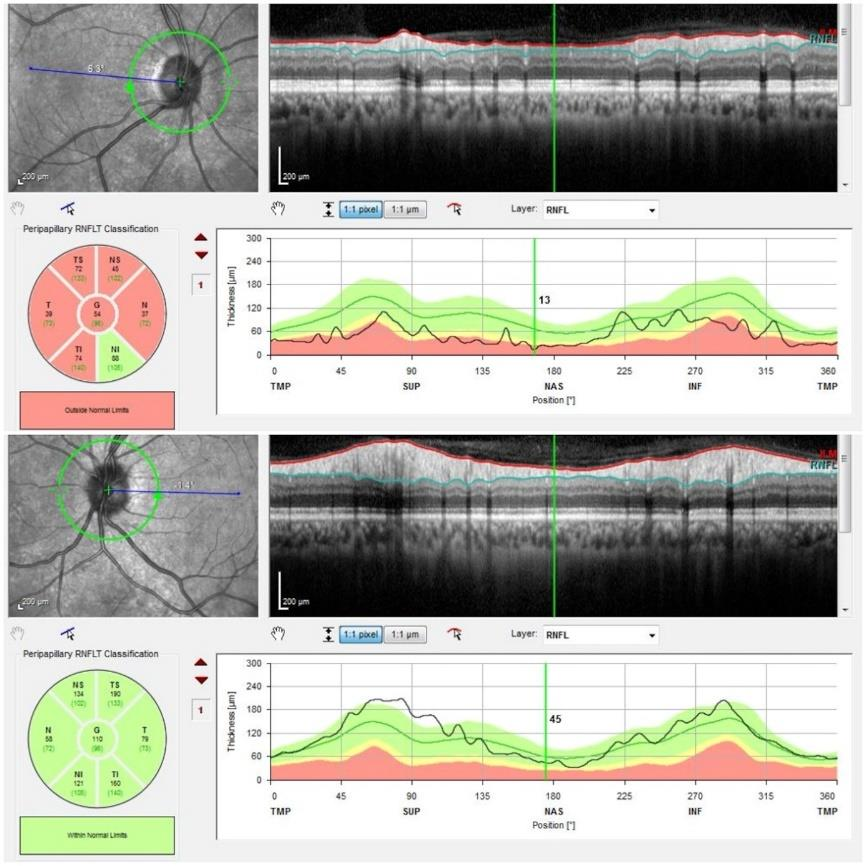
Widespread peripapillary RNFL thinning in the right eye and normal range of thickness in the left eye

**Fig. 2:**
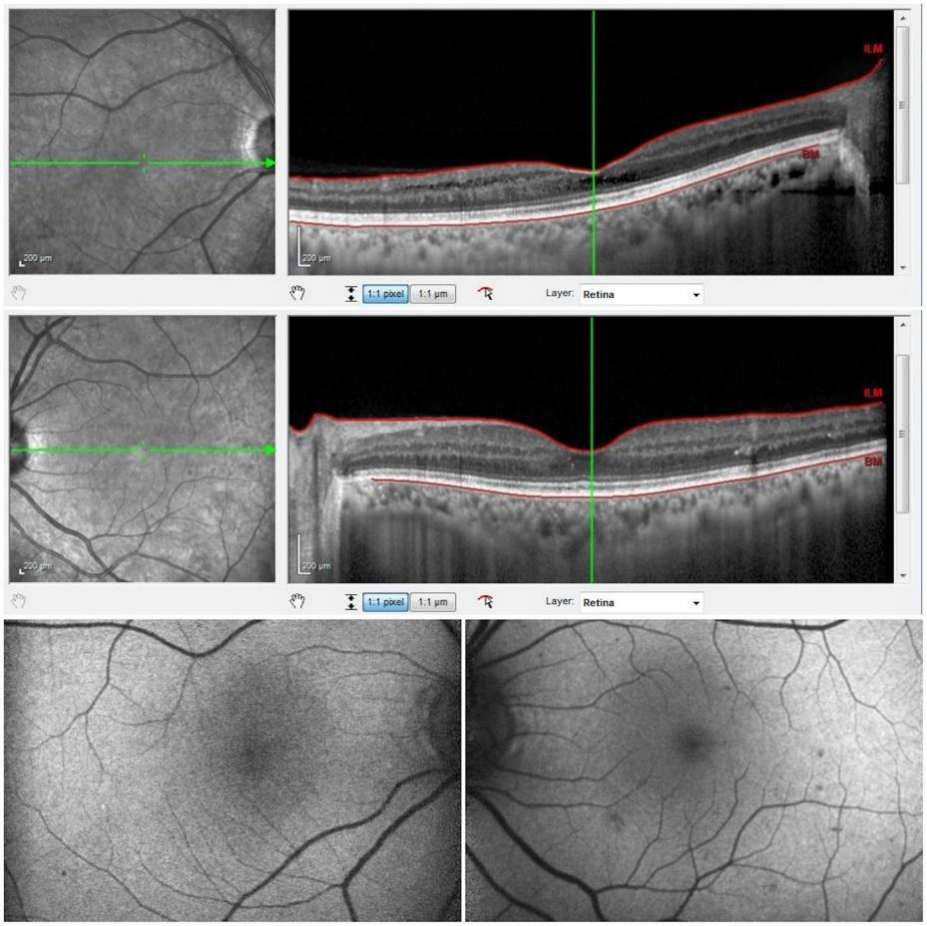
Retinoschisis and microcyst formations at the ONL in the central macula and additional retinoschisis formation at the INL in the temporal macula of the right eye, and normal macula OCT in the left eye

Fundus autofluorescence, macula OCT, and peripapillary RNFL thickness evaluations were made using a spectral-domain OCT (Spectralis; Heidelberg Engineering, Heidelberg, Germany). Scattered hyperautofluorescent patches were seen in both the right and the left eye. A widespread peripapillary RNFL thinning was seen in the right eye, while it was in the normal range of thickness in the left eye (**Fig. 1**). Retinoschisis and microcyst formations at the outer nuclear layer (ONL) in the central macula and additional retinoschisis formation at the inner nuclear layer (INL) in the temporal macula were observed in the macula OCT evaluation of the right eye (**Fig. 2**). The normal macula OCT examination was observed in the left eye (**Fig. 2**). The patient offered a written informed consent to share her medical records and photographs for academic purposes.

## Discussion

The studies investigating the peripapillary RNFL thickness demonstrated decreased RNFL in course of NAION [**7**,**8**]. Garcia-Basterra et al. [**9**] reported some NAION-associated OCT changes including the RNFL thickening at presentation and gradually decreasing during the next three months, and thinner inner plexiform layer and ganglion cell complex throughout the disease. In this case report, a widespread thinning in peripapillary RNFL was observed. This finding is not unexpected, especially when considering the patient’s 12-months history of decreased visual acuity. The most unique finding, in this case, was the demonstration of bilayered retinoschisis. The peripheral retina outer plexiform layer is commonly the affected retinal layer in the acquired retinoschisis [**10**]. In this patient, the ONL was affected in the central macula, while the INL was affected in the temporal side of the macula. Bilayered patterns and the localizations in the retinal structure in this patient were completely different from the cases presented in the literature.

Van Buren [**11**] described optic nerve or chiasm lesions-associated “cystic degeneration” due to trans-synaptic degeneration in the INL. Gills and Wadsworth [**12**] reported a cell population decrease in the INL in subjects with persistent low vision due to traumatic or compressive optic nerve lesions. These two reports demonstrated a possible relationship between NAION and retinal splitting. Gelfand et al. [**13**] reported that microcysts are related to patient’s older age, the disease’s longer course, and worse disability [**13**]. They also stated that microcysts should not be treated because of their low potential to cause macular edema [**13**]. The absence of significant central macular edema related to the splitting in the ONL in the central macula was another important finding of the case in this study.

Barboni et al. [**14**] disagreed with the trans-synaptic degeneration-related microcyst formation hypothesis. Their rationale is that microcysts occur in a few patients while trans-synaptic degeneration occurs in all patients [**14**]. A combination of both vitreous traction and optic atrophy is thought of as another mechanism [**15**]. Loss of ganglion cells and RNFL may cause the vitreous traction directly on the INL, resulting in retinoschisis [**15**]. Probably, vitreoretinal traction is an important factor for the emergence of microcysts like the trans-synaptic degeneration mechanism. 

## Conclusion

This study reported an unusual co-existence of NAION and bilayered retinoschisis formation, which affected the INL and ONL of the macula. The clinicians should be informed of the possible effects of NAION on the macula and further studies should be designed to evaluate macula with different imaging technologies.

**Conflict of Interest statement**

The authors state no conflict of interest.

**Informed Consent and Human and Animal Rights statement**

Informed consent has been obtained from all individuals included in this study.

**Authorization for the use of human subjects**

Ethical approval: The research related to human use complies with all the relevant national regulations, institutional policies, is by the tenets of the Helsinki Declaration, and has been approved by the review board of Hatay Education and Research Hospital, Hatay, Turkey.

**Acknowledgments**

None.

**Sources of Funding**

None.

**Disclosures**

None.
